# Immittance Studies of Bi_6_Fe_2_Ti_3_O_18_ Ceramics

**DOI:** 10.3390/ma13225286

**Published:** 2020-11-22

**Authors:** Agata Lisińska-Czekaj, Dionizy Czekaj, Barbara Garbarz-Glos, Wojciech Bąk

**Affiliations:** 1 Faculty of Mechanical Engineering, Gdańsk University of Technology, 11/12, Narutowicza St., 80-233 Gdańsk, Poland; dionizy.czekaj@pg.edu.pl; 2 Institute of Technology, Pedagogical University of Cracow, 2 Podchorążych Str., 30-084 Kraków, Poland; barbara.garbarz-glos@up.krakow.pl (B.G.-G.); wojciech.bak@up.krakow.pl (W.B.); 3 Institute of Technology, The Jan Grodek State University in Sanok, 6 Reymonta Str., 38-500 Sanok, Poland

**Keywords:** electroceramics, immittance spectroscopy, specific conductivity, activation energy

## Abstract

Results of studies focusing on the electric behavior of Bi_6_Fe_2_Ti_3_O_18_ (BFTO) ceramics are reported. BFTO ceramics were fabricated by solid state reaction methods. The simple oxides Bi_2_O_3_, TiO_2_, and Fe_2_O_3_ were used as starting materials. Immittance spectroscopy was chosen as a method to characterize electric and dielectric properties of polycrystalline ceramics. The experimental data were measured in the frequency range Δ*ν* = (10^−1^–10^7^) Hz and the temperature range Δ*T* = (−120–200) °C. Analysis of immittance data was performed in terms of complex impedance, electric modulus function, and conductivity. The activation energy corresponding to a non-Debye type of relaxation was found to be *E*_A_ = 0.573 eV, whereas the activation energy of conductivity relaxation frequency was found to be *E*_A_ = 0.570 eV. An assumption of a hopping conductivity mechanism for BFTO ceramics was studied by ‘universal’ Jonscher’s law. A value of the exponents was found to be within the “Jonscher’s range” (0.54 ≤ *n* ≤ 0.72). The *dc*-conductivity was extracted from the measurements. Activation energy for *dc*-conductivity was calculated to be *E*_DC_ = 0.78 eV, whereas the *dc* hopping activation energy was found to be *E*_H_ = 0.63 eV. The obtained results were discussed in terms of the jump relaxation model.

## 1. Introduction

Magnetoelectric multiferroics, which are simultaneously ferroelectric and ferromagnetic (or at least show some kind of magnetic ordering), present a somewhat obscure but tempting, from a scientific point-of-view, class of materials. Such materials have all the potential applications of both their parent ferroelectric and ferromagnetic materials. The emerging possibility of controlling the electrical properties by changing the magnetic field and controlling the magnetic properties by changing the electric field opens up new, broad horizons in the device’s design. For instance, memory elements in which data is stored both in the electric and the magnetic polarizations, or novel memory media, might allow the writing of a ferroelectric data bit and the reading of the magnetic field generated by association [1].

Let us consider bismuth ferrite BiFeO_3_ (BFO) – bismuth titanate (Bi_4_Ti_3_O_12_) compounds. BiFeO_3_ is known to exhibit a perovskite-type structure and is one of only a few materials in which (anti)ferromagnetism and ferroelectricity coexist at room temperature—which is one of the most desirable features for multiferroics. Therefore, being the only single-phase ABO_3_-type perovskite compound which possesses multiferroic properties at room temperature, BFO is considered to be the most promising candidate among various multiferroic materials for applications in the next generation memories and spintronics [2]. Bismuth titanate (Bi_4_Ti_3_O_12_) is a bismuth layer-structured ferroelectric material which was discovered by Aurivillius [3]. It is commonly used for various electronic industry applications like capacitors, transducers, non-volatile ferroelectric memory devices, and high temperature piezoelectric sensors. Combining these two materials, namely, bismuth titanate and bismuth ferrite exhibiting different physical properties, one can create novel materials. Thus, achieving rich functionality, Aurivillius phases of the Bi_4_Ti_3_O_12_-BiFeO_3_ system are known to combine ferroelectric, semiconducting, and ferromagnetic properties and they are potentially attractive for producing high-performance ceramics for information processing and information storage applications [4,5].

The Bi_6_Fe_2_Ti_3_O_18_ compound has been already synthesized either in a form of bulk polycrystalline ceramics or in a form of thin film. The synthesis of Aurivillius phases has usually been performed by the mixed oxide method, e.g., [6,7,8], but the molten salt method [9], hydrothermal method [10] and hot pressing method [11] were also utilized. Thin films of Bi_6_Fe_2_Ti_3_O_18_ were deposited by chemical solution deposition [12,13] or metalorganic decomposition [14] on different substrates (e.g., N-type Si wafers with (111) orientation [14], as well as (111) Pt/TiSiO_2_/Si and sapphire substrates [12,13] were utilized).

The present study was motivated by the high potential applications of Aurivilius phases of the Bi_4_Ti_3_O_12_-BiFeO_3_ system. Moreover, fundamental physics of multiferroic materials are rich and fascinating. Therefore, the ferromagnetic compound of the Bi_6_Fe_2_Ti_3_O_18_ (BFTO) composition was chosen as a material of investigation due to its high Curie temperature (*T* = 805 °C) and a second order magnetoelectric effect [10,15]. Although several studies on the impedance spectroscopy study of polycrystalline Bi_6_Fe_2_Ti_3_O_18_ have already been published, e.g., [6,11,12,13,16], the results are available for different objects (bulk ceramics or thin films), different methods of fabrication (bulk ceramics), or different conditions of the thin film crystallization. Moreover, the presented data are available for different temperatures or information on the crystal structure and the phase composition is missing, e.g., [6]. Present studies were focused on the dielectric response of polycrystalline BFTO multiferroic ceramics in the frequency range Δ*ν* = (10^−1^–10^7^) Hz and the temperature range Δ*T* = (−120–200) °C. The obtained results were discussed in terms of *ac*-conductivity as well as complex impedance and complex electric modulus formalisms.

## 2. Materials and Methods

BFTO ceramics were manufactured by the mixed oxide method (MOM). Bi_2_O_3_, TiO_2_, and Fe_2_O_3_ metal oxides (all 99.9% purity, Aldrich Chemical Co., Ltd., Merck KGaA, Darmstadt, Germany) were used for stoichiometric mixture preparation. After the calcination process (*T_calc_* = 720 °C), the pellets were formed and pressed into disks with a diameter of 10 mm and 1 mm thickness. Pressureless sintering was used for final densification of ceramic samples. The sintering temperature was *T_s_* = 980 °C, while the dwell time was *t*_s_ = 2 h. For thermal analysis measurements, the stoichiometric mixture of oxide powders, constituting the Bi_6_Fe_2_Ti_3_O_18_ composition, was used; whereas for X-ray diffraction analysis, the sintered BFTO ceramic sample was powdered to utilize advantages of the powder diffraction method. For dielectric spectroscopy measurements, the sintered ceramic disks were polished on both sides and covered with silver electrodes to form a disk-shaped parallel-plate capacitor.

The Alpha-AN High Performance Frequency Analyzer system combined with a cryogenic temperature control system (Quatro Cryosystem, Novocontrol Technologies GmbH & Co. KG, Montabaur, Germany) was used for broad-band dielectric spectroscopy measurements (BBDS) [17]. A strong point of BBDS is that using an appropriate strategy and various methods of analyzing experimental data, one is possible to separate the impact of individual electrically active areas (e.g., grains and grain boundaries) as well as characterize the dynamics of the electric charge both at the interfacial boundaries and inside the grains of the ceramic material [18,19]. BBDS is used for studying a fine nature of electrical behavior of electroceramic materials including ferroelectrics, solid electrolytes, and materials with mixed conductivity.

The measuring temperature range was Δ*T* = (−120 … + 200) °C, and the frequency range was Δ*ν* = (10^−1^ … 10^7^) Hz. Before the measurement, the system was cooled down with liquid nitrogen. The measurements were performed during the heating cycle. The impedance spectra were recorded 15 min after reaching the programmed temperature. The step size of the temperature change was Δ*T* = 5 °C. The WinDATA Novocontrol software was used for recording, visualization, and processing of experimental data. However, to check the consistency of the experimental data, a computer program by Boukamp was used [20]. Thus, the Kramers–Kronig test was carried out.

## 3. Results and Discussion

**Figure 1:**
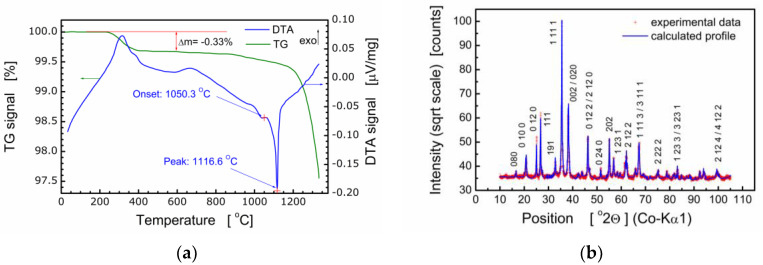
(**a**) Thermograms of the Bi_6_Fe_2_Ti_3_O_18_ (BFTO) powder; (**b**) X-ray diffraction pattern for BFTO compound.

It should be pointed out that bismuth layer-structured multiferroic compounds of the composition Bi_m+1_Fe_m–3_Ti_3_O_3m+3_ are difficult to produce because these compounds are formed in several stages and their thermal stability is low. Therefore, the process of fabrication of BFTO was controlled with both simultaneous thermal analysis (STA) and X-ray diffraction analysis. It was found by STA that the formation of the BFTO phase took place at the temperature range Δ*T* ≈ 650–700 °C [7]. For that reason, the temperature of calcination was chosen as *T_c_* = 720 °C. Results of the thermal analysis of BFTO powder are shown in Figure 1a.

Results of X-ray diffraction studies performed for ceramics sintered at *T_s_* = 850 °C, *T_s_* = 1080 °C [7], and *T_s_* = 980 °C [8,21] proved the essential influence of the sintering temperature on the crystal structure and phase composition of the Bi_6_Fe_2_Ti_3_O_18_ compound. It was found that the BFTO ceramics sintered at *T_s_* = 850 °C, *T_s_* = 1080 °C were multiphase ones and consisted of two phases, namely, the Aurivillius phase with *m* = 5 layers (i.e., the stoichiometric phase) and *m* = 4 (i.e., the phase with a reduced number of layers in the slab) [7]. Our earlier studies have shown [8,21] that Bi_6_Fe_2_Ti_3_O_18_ ceramics, sintered at *T_s_* = 980 °C, were single-phase and adopted the orthorhombic structure of the *Aba2* (41) space group. Therefore, for the purpose of the present studies, final sintering of BFTO ceramics was performed at *T_s_* = 980 °C. The resulting X-ray diffraction pattern is shown in Figure 1b.

Structural analyses based on the X-ray diffraction pattern (Figure 1b) show the BFTO compound crystallized in an orthorhombic structure, *Fmm2* space group, with the following elementary cell parameters: *a* = 5.4565(4) Å, *b* = 49.375(3) Å, and *c* = 5.4816(3) Å. The Rietveld refinement of the crystal structure was performed using the ICSD standard (ICSD collection code: 156257), primary reference [9]. The average size of crystallites was calculated 〈*D*〉 = 433.2 Å as well as the average strain 〈*ε*〉 = 0.043%. It is worth noting that quality parameters of the Rietveld refinement procedure were as follows: R(expected) = 2.81305%; R(profile) = 3.97795%; R(weighted profile) = 5.52957%; goodness of fit GOF = 3.86392%.

**Figure 2:**
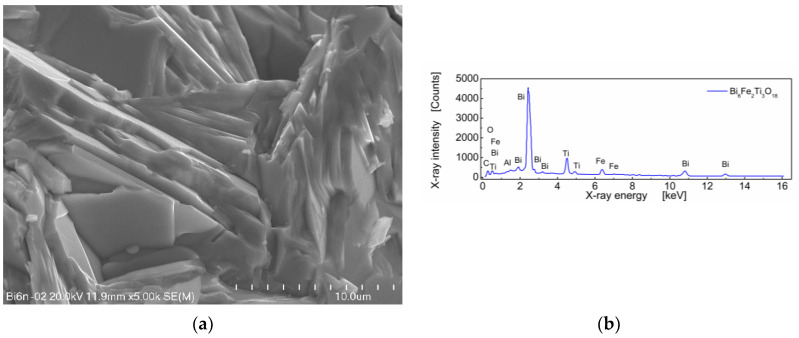
(**a**) Microstructure of BFTO ceramics; (**b**) the EDS spectrum for BFTO ceramics.

A SEM micrograph of sintered Bi_6_Fe_2_Ti_3_O_18_ ceramics and EDS spectrum is shown in Figure 2.

**Figure 3:**
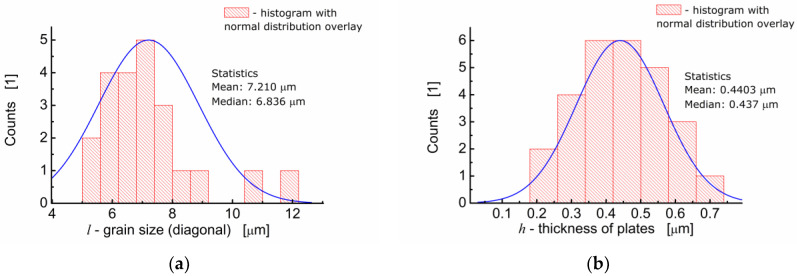
(**a**) Analysis of the grain size distribution for BFTO ceramics; (**b**) analysis of the thickness size distribution for plate-like grains of BFTO.

One can see in Figure 2a that BFTO grains adopt a plate-like shape. Measurements of the average grain size taken as an average diagonal of the plate-like grain were performed with the help of an ImageJ—A public domain Java image processing program [22]. It was found that the average grain size was 7.21 μm (Figure 3a). Apart from the diagonal, the thickness of the plates was measured. It was found that the average thickness of the plate-like grain was 0.44 μm. The resulting distribution of “thicknesses” of the plate-like grains is shown in Figure 3b.

The stoichiometric composition of the Bi_6_Fe_2_Ti_3_O_18_ compound expressed in the weight fraction of oxides is as follows: Bi_2_O_3_—77.7 wt%; Fe_2_O_3_—9.0 wt%; and TiO_2_—13.3 wt% [8]. The EDS quantitative results, calculated on the base of the spectrum shown in Figure 2b, for BFTO ceramics were as follows: Bi_2_O_3_—78.26 wt%; Fe_2_O_3_—8.80 wt%; and TiO_2_—12.94 wt%.

### 3.1. Complex Impedance Analysis

**Figure 4:**
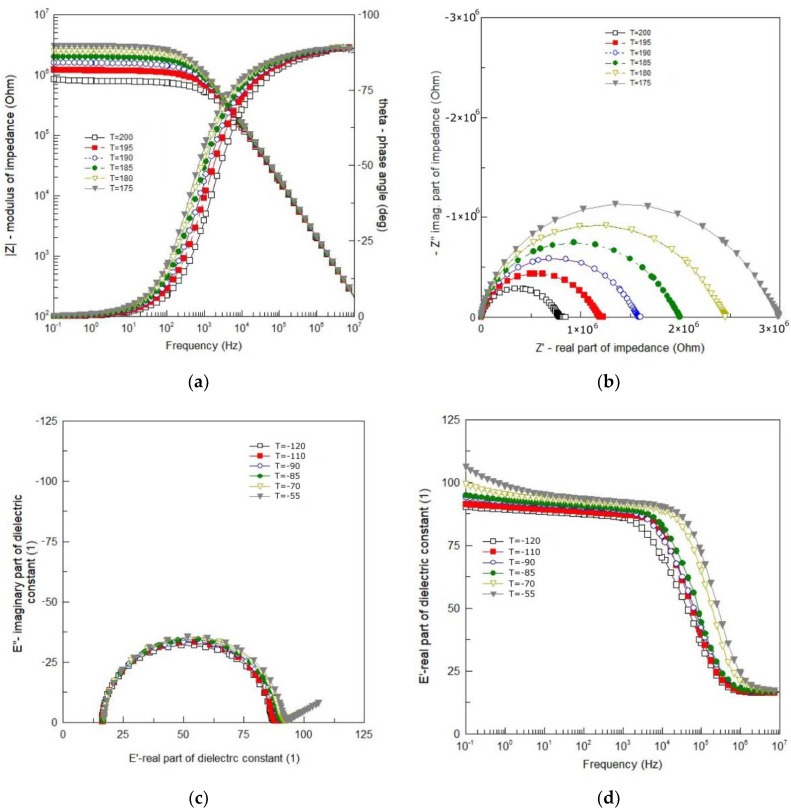
(**a**) Bode plot of impedance data; (**b**) complex impedance plot at 175 °C–200 °C; (**c**) Cole–Cole plot of impedance data at the temperature range from −120 °C to −55 °C; (**d**) spectroscopic plot of the real part of dielectric permittivity at −120 °C to −55 °C.

Dielectric spectroscopy is extremely susceptible to random disturbances that do not show up in the impedance spectrum at first glance. Therefore, in order to obtain reliable results as a result of the analysis of impedance data, it is necessary to test the consistency of the recorded measurement data. For this purpose, the Kramers–Kronig (K–K) equations were used to test the quality of measurement data. Inspection of the results showed high compliance of the measurement with the K–K calculations. The value of the “chi-square” parameter was obtained within the range *χ*^2^ = 5 × 10^−5^–2 × 10^−7^. Thus, an excellent quality of the measurements was confirmed. Results of the impedance measurements are shown in Figure 4.

One can see smooth and monotonic curves of modulus of the complex impedance |*Z*| that exhibit weak frequency dispersion in the low frequency range and strong frequency dependence in the high frequency range (Figure 4a). The changeover point from weak to strong dependence on frequency, shifts toward higher frequency with an increase in temperature. One can see in Figure 4a that in the high frequency range |*Z*| (*ν*) curves for different temperatures overlap each other. The curves representing a spectroscopic plot of the phase angle (*θ*) exhibit a sigmoidal shape (in a semi-logarithmic scale). They are also smooth and change monotonically from *θ* = 0° for low frequency to *θ* = −90° for high frequency (Figure 4a). They shift toward higher frequency with an increase in temperature.

The dependence of the imaginary part of complex impedance (−*Z*″) on the real part of complex impedance (*Z*′) is shown in Figure 4b. One can see that despite the isotropic linear scale used, the experimental curves (Figure 4b) are rarely ideal semicircles. In the present case, the curves resemble arcs (deformed, flattened semicircles) with the centers below the real axis. The frequency at which the arc reaches a maximum corresponds to the relaxation frequency, e.g., [16]:

(1) $$\omega_{m}\tau_{m}=1,$$

where *ω*_m_, *τ*_m_—relaxation frequency and relaxation time, respectively. As the temperature rises, the depression angle increases and the arc radius decreases, which indicates the thermal activation of the conduction mechanism. The shape of the Nyquist diagram (Figure 4b) shows that the relaxation time of the polarization processes taking place in BFTO ceramics cannot be defined as a single quantity, but as a quantity with a certain distribution around the mean value.

Impedance data for the temperature range Δ*T* = −120 °C–55 °C are shown in Figure 1 in a form of the Cole–Cole plot (Figure 4c) and the spectroscopic dependence of the real part of dielectric permittivity (Figure 4d). Almost ideal semicircles, shown in Figure 4c, mean that the phenomena responsible for relaxation in the BFTO ceramics can be described by a simple Debye model with one relaxation time. The main advantage of such presentation is a possibility to read from the plot values for both the static (*ε*_s_) and high frequency (*ε*_∞_) limits of dielectric permittivity. The phenomena of dielectric relaxation, however, become significantly more complicated with increasing temperature. As the temperature increases, a straight line appears on the Cole–Cole diagram (for low frequencies). Figure 4d demonstrates the frequency dependence of the real part (*ε*′) of the complex permittivity for the BFTO ceramics at various temperatures chosen from the temperature range Δ*T* = −120 °C–55 °C. It is clear from Figure 4d that the BFTO ceramics exhibit a low-frequency dispersion. However, what may attract our attention is the step-like decrease in *ε*′ that shifts to higher frequency with increasing the temperature, indicating the thermally activated mechanism.

**Figure 5:**
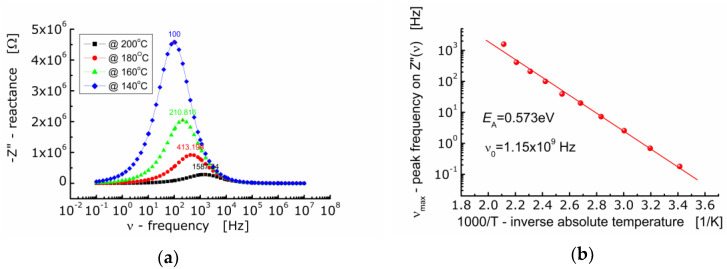
(**a**) Dependence of imaginary part of impedance (−*Z*″) on frequency for BFTO ceramics at 140 °C–200 °C; (**b**) dependence of frequency (*ν*_max_) corresponding to the maximum −*Z*″(*ν*) on inverse temperature.

Figure 5a shows the dependence of the imaginary part of impedance (−*Z*”) of BFTO ceramics on the frequency. One can see that as the temperature rises, the maximum of the *Z*″-curve shifts towards higher values of frequency. The curves taken at different temperatures are not symmetrical and wide (FWHM ≈ 2 decades of frequency). The dependence of *ν*_max_ on inverse absolute temperature is shown in Figure 5b. The activation energy corresponding to relaxation was found to be *E*_A_ = 0.573 eV.

Presentation of impedance data in the complex −*Z*″−*Z*′ plane (the so-called Nyquist diagram) or in the form of a spectroscopic dependence *Z*″(*ν*) (the so-called Bode diagram) allows to distinguish the contribution of the areas with the highest resistance to the total impedance response of the tested system. They are useful when the relaxation frequencies of polarization processes taking place inside the grains (*ν*_b_), at the grain boundaries (*ν*_gb_) and in the electrode areas (*ν*_e_), differ significantly (e.g., *ν*_b_ >> *ν*_gb_ >> *ν*_el_). However, they may not be sufficient to define the contribution of the areas comprising the interior of the grains (bulk) with highly resistive grain boundaries. They are also of little use when the contributions from individual areas of the ceramic microstructure (grains and grain boundaries) overlap (see: flattened, deformed arcs in Figure 4b). In such cases, it is advisable to represent the impedance data using an electric modulus function that is sensitive to input from low capacitance areas [23].

### 3.2. Electric Modulus Analysis

In dielectric spectroscopy, a function called the electric modulus is often used. This function is complex and is defined as the reciprocal of the dielectric constant:

(2) $$M*=\frac{1}{\varepsilon *}={\left(\varepsilon^{\prime }-j\varepsilon^{\prime \prime }\right)}^{-1}=M^{\prime }+jM^{\prime \prime },$$

where *ε*′, *ε*″—real and imaginary part of dielectric constant, respectively.

The electric modulus function is neither a directly measurable quantity nor directly related to any microscopic physical processes. However, it completes the set of four interrelated to each other electrical relaxation functions, which are complex permittivity, complex conductivity, complex resistivity, and complex electric modulus [24]. The advantages of an analysis based on the use of electrical modulus functions are apparent when there is a need to distinguish the electrode polarization phenomena or assess the relaxation time of electrical conductivity [25]. Presentation of the impedance data in the form of a spectroscopic dependence of the electric modulus often allows to emphasize the contribution of polarization phenomena occurring inside the grains (bulk) and characterized by the lowest capacitance. A contribution of other polarization processes exhibiting small differences in capacitance may also be revealed [23].

**Figure 6:**
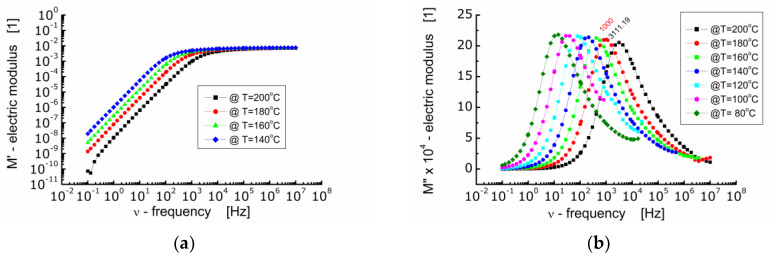
(**a**) Dependence of the real part of electric modulus (*M*′) on frequency; (**b**) dependence of the imaginary part of electric modulus (*M*″) on frequency at various temperatures for BFTO ceramics.

Figure 6 shows the dependence of the real part of electric modulus *M*′ (Figure 6a) and the imaginary part of electric modulus *M*″ (Figure 6b) on frequency at diverse temperatures for BFTO ceramics.

One can see from Figure 6a that in the low frequency range *M*′ reaches very low values. A drive of *M*′ towards zero points out that the force restoring the flow of charge decays as the frequency of the measuring electric field decreases. At the same time, it indicates that the electrode and/or interfacial processes, which usually manifest themselves within the low frequency range, do not significantly contribute to the total dielectric response of BFTO ceramics [26]. In the high frequency range, *M*′ tends to a constant value the same for all measurements carried out at different temperatures. This in turn represents the reciprocal value of the frequency independent dielectric constant characterizing the bound charge response. The changeover point from a strong to weak dependence of *M*′ on frequency shifts toward higher frequency with an increase in temperature.

The dependence of the imaginary part of the electric modulus on frequency presented in Figure 6b allows to highlight the relaxation processes taking place inside the grains (bulk). It can be seen that as the temperature increases, the maxima on *M*″ curves shift towards higher frequencies and the height of the maximum decreases slightly. It is worth noting that the dependence of *M*″ on frequency should be of the Lorentz type if the relaxation process is the exponential one. Nevertheless, a deviation from the Debye behavior of relaxation phenomena in BFTO ceramics has been noted. The Lorentz fit of the *M*″ spectroscopic dependence showed that the maxima are wide (FWHM was about 2 decades) and asymmetric. Two possible reasons for such behavior observed in BFTO ceramics can be assumed, namely, the presence of a distribution of relaxation times or stretching of the relaxation times [25].

**Figure 7:**
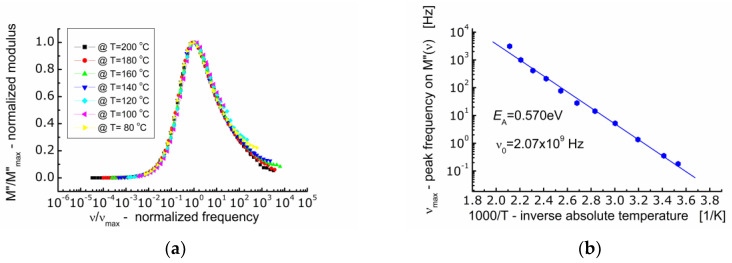
(**a**) Dependence of normalized modulus (*M*″*/M*″*_max_*) on so-called frequency (*ν/ν_max_*); (**b**) dependence of frequency (*ν*_max_) corresponding to the maximum *M*″(*ν*) on inverse temperature.

The spectroscopic *M*″ data were normalized along the ordinate axis and abscissa axis (Figure 7a). Normalization along the ordinate axis consisted in dividing the current values of *M*″ by the maximum value of the imaginary component of the electric modulus (*M*″*/M*_max_). Normalization along the abscissa axis consisted in dividing the current value of the measuring frequency by the frequency corresponding to the maximum value of the imaginary component of the electric modulus (*ν*/*ν*_max_) known as the conduction relaxation frequency (*ν*_max_). As a result of such data treatment, it was found that the profile of the spectroscopic curve of the electric modulus (*M*″) does not change with temperature. All the *M*″-curves were superimposed on one so-called primary curve.

The analysis of the dependence of the conduction relaxation frequency of BFTO ceramics on the inverse absolute temperature (Figure 7b) allowed to calculate the activation energy of the process *E*_A_ = 0.570 eV.

### 3.3. Electric Conductivity Analysis

**Figure 8:**
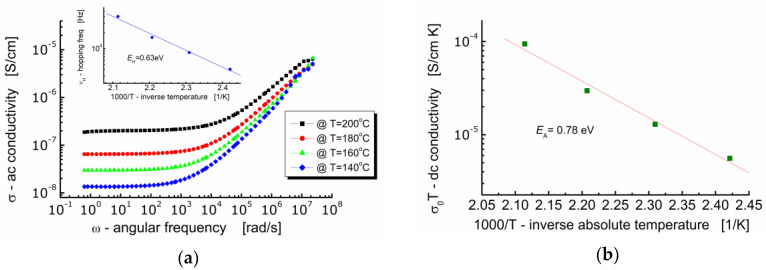
(**a**) Dependence of *ac*-conductivity on frequency. The insertion shows dependence of the hopping frequency on inverse absolute temperature; (**b**) dependence of *dc*-conductivity *σ*(0)*T* on inverse temperature.

Figure 8a shows the dependence of *ac*-conductivity (*σ*) on angular frequency (*ω*) at various temperatures for Bi_6_Fe_2_Ti_3_O_18_ ceramics. One can see in Figure 8a that the behavior of BFTO ceramics is typical for ionic materials. It can be seen that in the low-frequency region, the electrical conductivity hardly depends on the frequency (plateau is present) and takes a value equal to the volume conductivity of the sample *σ*(0). With an increase in the measuring frequency, a strong high-frequency dependence of the electric conductivity of the sample appears. On the other hand, the *dc*-plateau is temperature dependent—the raise of conductivity is observed while temperature increases. The plot in Figure 8b shows the dependence of conductivity *σ*(0)*T* (assuming a hopping conduction mechanism) on 1000/*T* (*T* is an absolute temperature).

The expression “hopping” refers to the sudden movement of an electric charge carrier from one location to another in its vicinity. The process of electric current conduction based on the hopping mechanism does not take place through the conduction band. It occurs through the use of localized energy states within the band gap and it is characteristic for dielectrics, like, e.g., BFTO ceramics.

It can be seen in Figure 8a that the conductivity spectroscopic curve bent strongly starting from a characteristic point. At that bent point, not only the marked dependence of the conductivity on frequency begins, but also the relaxation of the conductivity starts. Geometrically, it can be found as the intersection of two tangent lines to the conductivity spectroscopic curve, namely, one tangent plotted in the low frequency region and the other tangent plotted in the high frequency region. The frequency that corresponds to that characteristic point is referred as a hopping rate (*ν*_H_), e.g., [27]. Values of the hopping rate for BFTO ceramics were read out from Figure 8a and plotted against inverse absolute temperature (the insertion in Figure 8a).

The *dc*-conductivity activation energy was found to be *E*_DC_ = 0.78 eV, whereas the hopping activation energy was found to be *E*_H_ = 0.63 eV. This result suggests that the charge carriers have to overcome the different energy barriers while conducting or relaxing [28]. It can be explained in terms of a jump relaxation model [29] which allows for two competing relaxation processes. The first is the forward-backward hop of the ion. The second is the relaxation of the crystal lattice in the immediate vicinity of the ion and the creation of a new energetically convenient vacant location for the ion to hop.

For most ceramic materials, the dependence of ionic conductivity on frequency can be described by Jonscher’s law of dielectric response [30] as

(3) $$\sigma (\omega )=\sigma (0)+A\omega^{n},$$

where *σ*(0) is the *dc*-conductivity, *A* is a constant, and the exponent *n* is within 0 < *n* < 1. Experimental data were subjected to the fitting procedure according to Equation (3).

One can see from Table 1 that fitting quality parameters, namely, *χ*^2^ (chi-squared) and *R*^2^ (r-squared), state that the fitting quality improves with increasing temperature.

**Figure 9:**
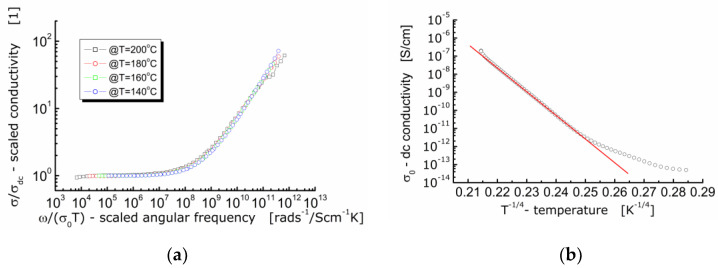
(**a**) Conductivity master curve of BFTO ceramics; (**b**) dependence of *dc*-conductivity on the reciprocal of the fourth root of the absolute temperature for BFTO ceramics.

It is worth noting that conductivity is a fundamental physical quantity. Therefore, presentation and analysis of the dielectric data of ionic conductors using the concept of *ac*-conductivity is preferable. For that reason, an attempt was made to scale the experimental data of *ac*-conductivity according to the approach given in the literature [31]. The results are shown in Figure 9a.

It can be seen in Figure 9a that the curves measured at different temperatures and showing the dependence of the normalized specific *ac*-conductivity (*σ*/*σ*_DC_) on the circular frequency divided by *σ*(0)*T* (i.e., ionic conductivity for hopping mechanism) lie on the same primary curve. The fact that the *ac*-conductivity curves recorded at different temperatures are superimposed and their profile remains unchanged, allows us to conclude that the conductivity relaxation mechanism is independent on temperature [25]. An experimental confirmation for conduction taking place according to the hopping mechanism is the linear dependence of conductivity, or electric resistivity in relation to the absolute temperature raised to the power (−1/4). One can see in Figure 9b that the linear dependence is present but for temperature higher than *T* ≈ −17 °C. Below that temperature, the dependence can hardly be fitted with a linear function exhibiting the same slope.

Considering the existence of structural defects such as oxygen deficiency, bismuth volatilization, and imperfect arrangement of Ti and Fe ions in the crystal lattice of BFTO ceramics valence transfer may occur between Fe^3+^ and Fe^2+^ in order to maintain the electrical neutrality of the system—as reported in literature [12,32]. Therefore, one may suppose that the relaxation was due to the hopping electron between Fe^2+^ and Fe^3+^, executing short-range movement. However, taking into consideration the atoms’ positions in the orthorhombic structure, *Fmm2* space group [9], one can see that when Fe ions are located at Ti^4+^ sites, the next neighbor oxygen can be vacant forming a neutral center which relaxes the hopping conduction ions.

## 4. Conclusions

Almost the same value of activation energies were obtained from the analyses of −*Z*″ and *M*″ spectroscopic data. The activation energy corresponding to non-Debye type of relaxation calculated on the base of the complex impedance analysis was found to be *E*_A_ = 0.573 eV. On the base of complex electric modulus formalisms, the activation energy of conductivity relaxation frequency was found to be *E*_A_ = 0.570 eV. The *ac*-conductivity data of BFTO multiferroic ceramics were found to obey Jonscher’s universal power law of dielectric response. A value of the exponents was 0.54 ≤ *n* ≤ 0.72. The hopping frequency was found to be temperature dependent and the hopping activation energy was found to be *E*_H_ = 0.63 eV. On the base of conductivity approach, the *dc*-conductivity activation energy was extracted from the frequency independent plateau region. It was found to be *E*_DC_ = 0.78 eV. The *dc*-hopping mechanism was confirmed with the linear dependence of conductivity on the inverse fourth root of temperature (in Kelvin). A difference between the hopping activation energy (*E*_H_) and the *dc*-conductivity activation energy (*E*_DC_) can be ascribed to two competing mechanisms of relaxation appropriate for the jump relaxation model.

## Figures and Tables

**Table 1:** Results of fitting the experimental data to the Jonscher equation.

Temperature, [°C]	*σ*(0) × 10^8^, [Scm^−1^]	Exponent *n*, [1]	A × 10^11^, [Scm^−1^rad^−n^]	*χ*^2^ × 10^15^, [1]	R^2^, [1]
140	1.3523	0.69896	4.0590	17.007	0.98101
160	2.9900	0.72308	2.9343	7.9279	0.99482
180	6.5479	0.53998	53.609	4.9587	0.99605
200	19.928	0.58696	40.520	1.3978	0.99917
